# Synthesizing developmental trajectories

**DOI:** 10.1371/journal.pcbi.1005742

**Published:** 2017-09-18

**Authors:** Paul Villoutreix, Joakim Andén, Bomyi Lim, Hang Lu, Ioannis G. Kevrekidis, Amit Singer, Stanislav Y. Shvartsman

**Affiliations:** 1 Lewis-Sigler Institute for Integrative Genomics, Princeton University, Princeton, New Jersey, United States of America; 2 Program in Applied and Computational Mathematics, Princeton University, Princeton, New Jersey, United States of America; 3 Department of Chemical and Biological Engineering, Princeton University, Princeton, New Jersey, United States of America; 4 School of Chemical and Biomolecular Engineering, Georgia Institute of Technology, Atlanta, Georgia, United States of America; 5 Interdisciplinary Program in Bioengineering, Georgia Institute of Technology, Atlanta, Georgia, United States of America; 6 Department of Mathematics, Princeton University, Princeton, New Jersey, United States of America; Pennsylvania State University, UNITED STATES

## Abstract

Dynamical processes in biology are studied using an ever-increasing number of techniques, each of which brings out unique features of the system. One of the current challenges is to develop systematic approaches for fusing heterogeneous datasets into an integrated view of multivariable dynamics. We demonstrate that heterogeneous data fusion can be successfully implemented within a semi-supervised learning framework that exploits the intrinsic geometry of high-dimensional datasets. We illustrate our approach using a dataset from studies of pattern formation in *Drosophila*. The result is a continuous trajectory that reveals the joint dynamics of gene expression, subcellular protein localization, protein phosphorylation, and tissue morphogenesis. Our approach can be readily adapted to other imaging modalities and forms a starting point for further steps of data analytics and modeling of biological dynamics.

## Author summary

A wide range of problems in biology require analysis of multivariable dynamics in space and time. As a rule, the multiscale nature and complexity of real systems precludes simultaneous monitoring of all the relevant variables, and multivariable dynamics must be synthesized from partial views provided by different experimental techniques. We present a formal framework for accomplishing this task in the context of imaging studies of pattern formation in developing tissues.

## Introduction

The need to synthesize data from different observations into coherent multivariable trajectories is discussed in multiple contexts, from physics to social sciences, but systematic approaches for accomplishing this task have yet to be established [1–5]. Here we address this task for imaging studies of developing tissues, where patterns of cell fates are established by complex regulatory networks [6–8]. Advances in live imaging continue to provide new insights into the dynamics of individual components in these networks, but imaging more than three reporters at the same time is still challenging and limited to model genetic organisms [9, 10]. Furthermore, in the absence of reliable live reporters, dynamics of some state variables can only be inferred from fixed tissues. Because of these limitations, extracting the multivariable dynamics from the heterogeneous datasets collected by imaging of live and fixed tissues becomes a non-trivial task [11, 12].

**Fig 1:**
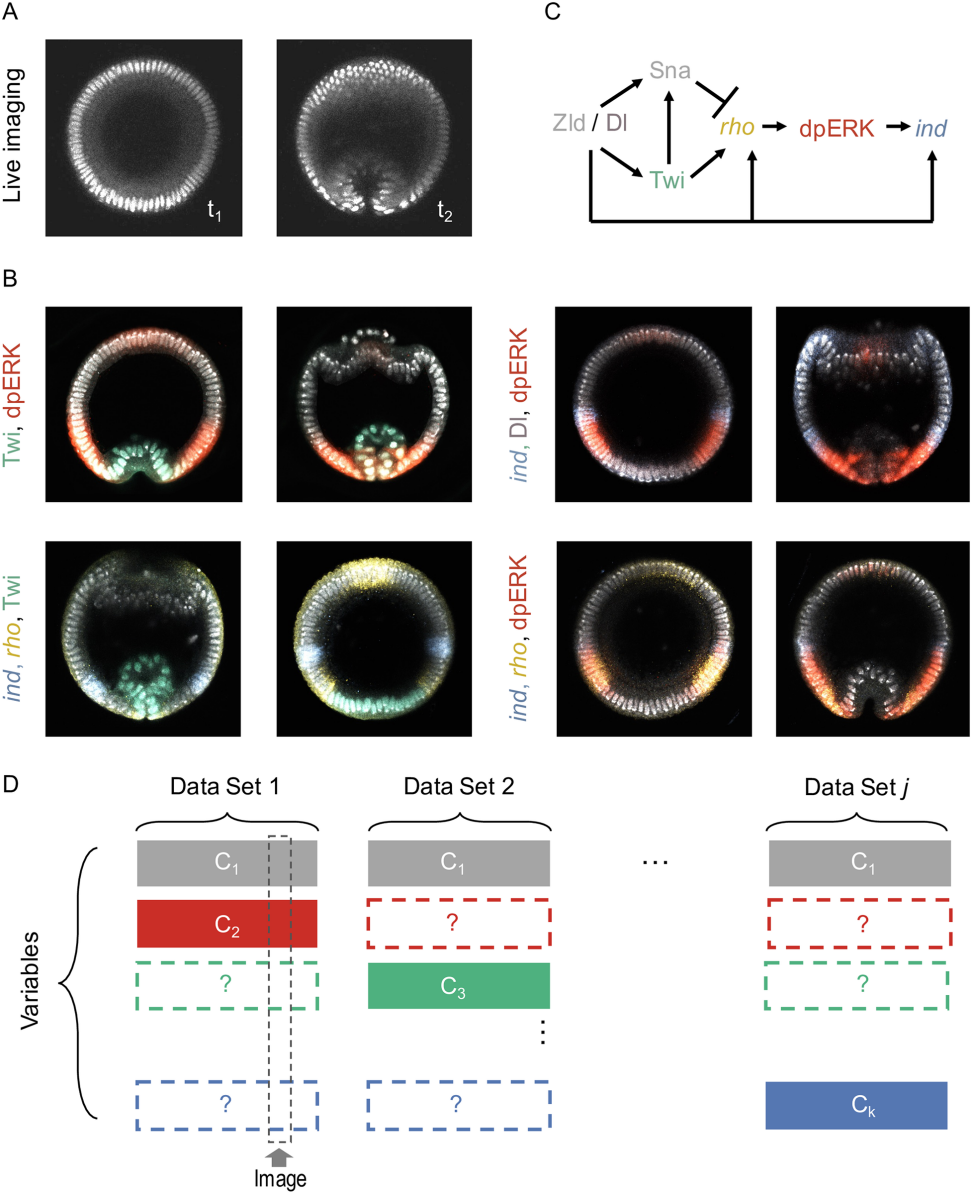
Stating the problem of data fusion. (A-B) Example datasets of molecular signals and morphology during the DV patterning of *Drosophila* embryo; all images are collected from optical cross-section along the DV axis, ∼ 15% from the posterior pole of the embryo. (A) Frames from a live imaging movie, showing positions of nuclei during the early stages of gastrulation. (B) Images of fixed embryos, stained with probes and antibodies revealing the spatial patterns of nuclear Dl (pink), Twi (green), dually phosphorylated ERK (red), and transcripts of *rho* (yellow) and *ind* (blue). (C) A fragment of a DV patterning network in the early *Drosophila* embryo. (D) Data fusion as a matrix completion problem: Each row corresponds to a variable, e.g. nuclear positions, gene expression levels, time stamp, revealed by visualizing different molecular or cellular components, nuclei, transcripts, or protein phosphorylation. Each column of the matrix corresponds to an image giving access to some of the states through various channels. The remaining states, labeled with a question mark, must be estimated from other datasets.

The problem can be illustrated by an imaging dataset from the early *Drosophila* embryo (Fig 1A and 1B), a model system in which a graded profile of the nuclear localization of transcription factor Dorsal (Dl) establishes the dorsoventral (DV) stripes of gene expression that control cell fates and tissue deformations [13–15]. Current mechanisms of the DV patterning system invoke multiple state variables, such as the levels of gene expression and protein phosphorylation [16] (Fig 1C). These mechanisms were elucidated in studies that reveal only a small subset of the full state space, most commonly 2-3 variables per experiment. Can these partial views be fused into a consistent multivariable trajectory? This is a general question that applies to essentially all developmental systems.

We realized that this question can be addressed by casting the task of data fusion as a matrix completion problem (Fig 1D). Specifically, an image of a fixed embryo or a frame from a live imaging movie can be viewed as a column in a matrix where rows correspond to the relevant variables, such as developmental time or the level of gene expression at a given position. Because of limitations in the number of states that can be accessed simultaneously, the matrix is incomplete. For example, live imaging of gastrulation provides information about nuclear positions as a function of time, but is silent about the levels of gene expression. On the other hand, an image of a fixed embryo reveals the distribution of an active enzyme but has no direct temporal information. Thus, multivariable data fusion requires completing this matrix, filling in the missing components by estimates informed by the rest of the data. Below we show how this task can be accomplished by solving a suitably posed semi-supervised learning problem. We first provide a closed-form solution to this problem and then demonstrate its successful performance on synthetic and experimental datasets.

## Results

### Semi-supervised learning framework for matrix completion

We assume here that all experiments contain a common variable, which is sufficient to determine all other variables that can be measured or to be predicted. For instance, this variable is revealed by a signal that reports positions of nuclei. This means that the first row in the matrix is complete. To complete other rows, we must establish the mappings between the common variable and each of the target variables. These mappings can be found within a semi-supervised learning framework, in which the values of the variables in the incomplete rows are estimated from a training dataset [17, 18].

**Fig 2:**
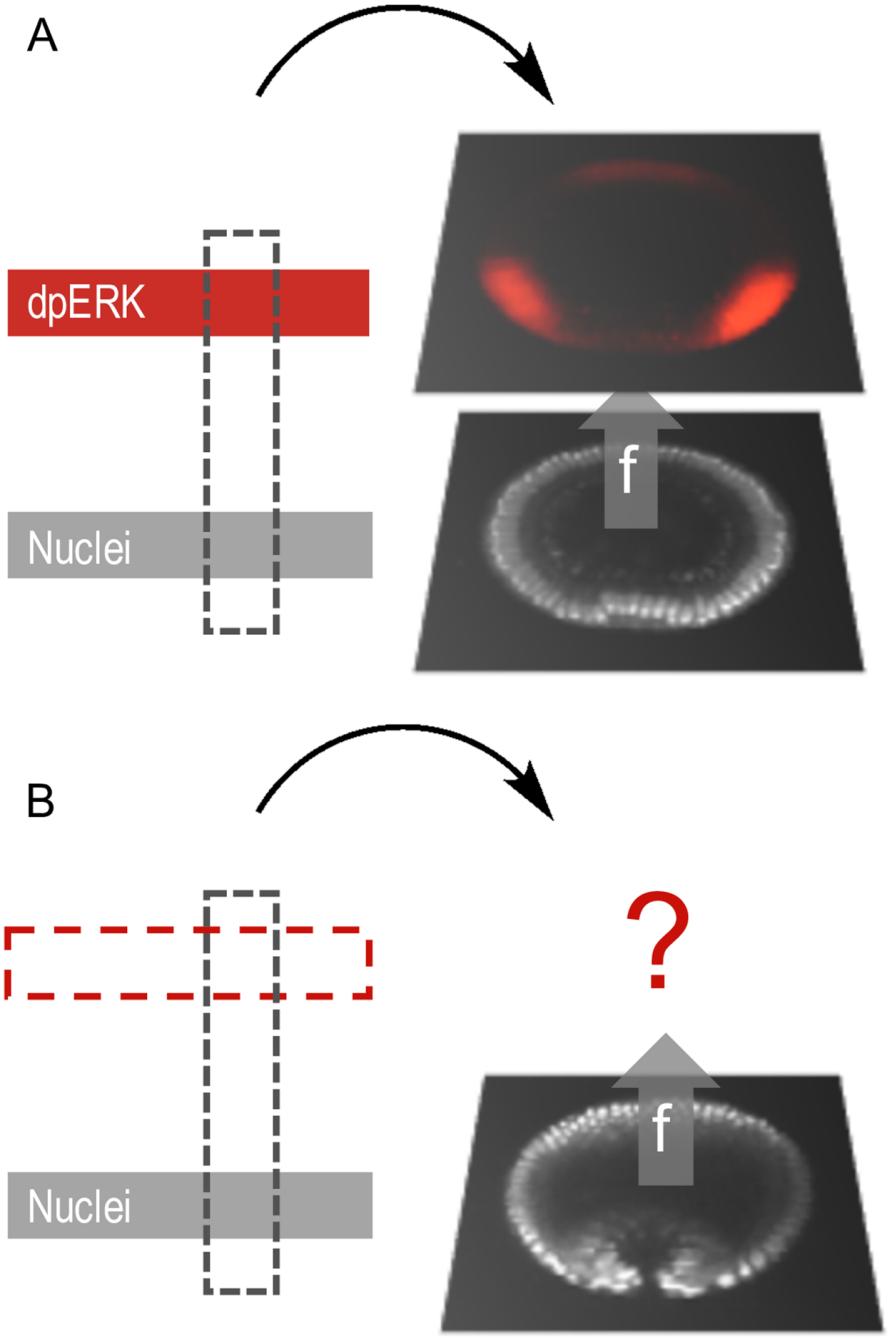
Learning a mapping from a common channel. An experimental image can be decomposed into various channels. E.g., red: dpERK, visualized with a phosphospecific antibody, gray: nuclei, visualized through either DAPI (in fixed images) or Histone-RFP (in live imaging). The training ensemble of labeled images (A) is used to predict the labels on a set of unlabeled images (B) using common information, the morphology obtained through the nuclei signal in this case. Morphological proximity yields similar labels.

As an example, consider images from fixed embryos that are stained to reveal the spatial pattern of an active enzyme, visualized using a phosphospecific antibody (Fig 2). They provide labeled data points that contain information about the common variable and a specific target variable. On the other hand, images without this staining, such as the frames from live imaging of morphogenesis, provide unlabeled data points with only the common variable. By finding a mapping between the common and target variables, we can essentially “color” the frames of a live imaging movie by snapshots of molecular patterns from fixed embryos.

A critical assumption in finding the mappings is that the multivariable dynamics of the patterning process are both low-dimensional and smooth with respect to the underlying parameters. This assumption is supported by studies with mathematical models of specific biological systems and by computational analysis of datasets from imaging studies of development [19, 20]. More formally, we consider a set of data points (*x*_1_, …, *x*_*l*_, *x*_*l*+1_, …, *x*_*l*+*u*_) belonging to a space X. These points correspond to the values of the common variable in the complete row. On the other hand, a row corresponding to any one of the target variables is incomplete. The values in the filled columns of this row are called labels. These are denoted (*y*_1_, …, *y*_*l*_) and belong to a target space Y. The semi-supervised learning techniques transfer the information contained in the labeled data points ((*x*_1_, *y*_1_), …, (*x*_*l*_, *y*_*l*_)) to the unlabeled points (*x*_*l*+1_, …, *x*_*l*+*u*_), while preserving the intrinsic structure of the dataset [18]. Stated otherwise, these techniques *learn* the mapping *y* = *f*(*x*) assuming that the considered process is smooth, which means that similar values of *x* give rise to similar values of *f*(*x*).

The missing values, corresponding to the unlabeled data points in each of the incomplete rows, are found by solving the following optimization problem:

$$\begin{array}{c} \vec{f}=\underset{\begin{array}{c} \vec{f}\in Y^{l+u}\\ \forall i\leq l,f_{i}=y_{i} \end{array}}{argmin}\sum_{i,j=1}^{l+u}w_{i,j}∥f_{i}-f_{j}{∥}^{2} \end{array}$$ (1)

where $\vec{f}=\left(f_{1},...,f_{l+u}\right)$ are the values of the target variable on the data points (*x*_1_, …, *x*_*l*+*u*_), the considered norm in Y is the Euclidean distance and the weights *w*_*i*,*j*_ represent the similarity between two data points *x*_*i*_ and *x*_*j*_. The norm in the space X can for example be the Euclidean distance in the space where each dimension corresponds to an image pixel or some coordinate in an arbitrary feature transform of that image. Other distances are possible. For example, the one-norm (or *L*^1^ distance) can be used, which increases robustness to outliers in the data. That being said, as long as these distances preserve the low-dimensional manifold structure, they will yield similar results as the number of points goes to infinity.

This quadratic optimization problem, known as harmonic extension, has a unique solution that relates the unlabeled data points *f*_*l*+1_, …, *f*_*l*+*u*_ to the labels *y*_1_, …, *y*_*l*_ where Y=R [17, 21]. The explicit solution reads:

$$\begin{array}{c} {\vec{f}}^{u}={\left(D_{u}-W_{uu}\right)}^{-1}W_{ul}Y \end{array}$$ (2)

where *Y* = (*y*_1_, …, *y*_*l*_) and ${\vec{f}}^{u}=\left(f_{l+1},...,f_{l+u}\right)$, and $d_{i}=\sum_{j=1}^{l+u}w_{i,j}$, *D*_*u*_ = diag(*d*_*l*+1_, …, *d*_*l*+*u*_), *W*_*uu*_ = (*w*_*i*,*j*_)_*l*+1≤*i*,*j*≤*l*+*u*_, and $W_{ul}={\left(w_{i,j}\right)}_{\begin{array}{c} l+1\leq i\leq l+u\\ 1\leq j\leq l \end{array}}$ (S1 Text).

### Illustrative example

To illustrate our method, we considered a one-dimensional nonlinear trajectory in a three-dimensional space. The trajectory is given by the set of equations

$$\begin{array}{c} \{\begin{array}{cc} x^{\left(1\right)}\left(t\right) & =at\;\left(\text{cos}\left(bt\right)+\epsilon^{\left(1\right)}\right)\\ x^{\left(2\right)}\left(t\right) & =at\;\left(\text{sin}\left(bt\right)+\epsilon^{\left(2\right)}\right)\\ y\left(t\right) & =ct\;\text{exp}\left(-d{\left(t-e\right)}^{2}\right) \end{array} \end{array}$$ (3)

where *a*, *b*, *c*, *d*, *e* are constants, *ϵ*^(1)^ and *ϵ*^(2)^ are Gaussian noise sources and *t* is a real-valued parameter. The set of points (*x*^(1)^(*t*), *x*^(2)^(*t*)) forms a one-dimensional non-linear manifold embedded in the two dimensional plane and it is parameterized by *t*. These points are analogs of the embryo morphology. In the absence of noise, this mapping from *t* to the 2D plane can be inverted as $t=\frac{1}{\left|a\right|}\sqrt{{\left(x^{\left(1\right)}\right)}^{2}\left(t\right)+{\left(x^{\left(2\right)}\right)}^{2}\left(t\right)}$. The signal *y*(*t*) is a smooth function of *t* and is thus a smooth function of (*x*^(1)^, *x*^(2)^) by composition. In this example, *y* corresponds to the target modality that we would like to estimate.

**Fig 3:**
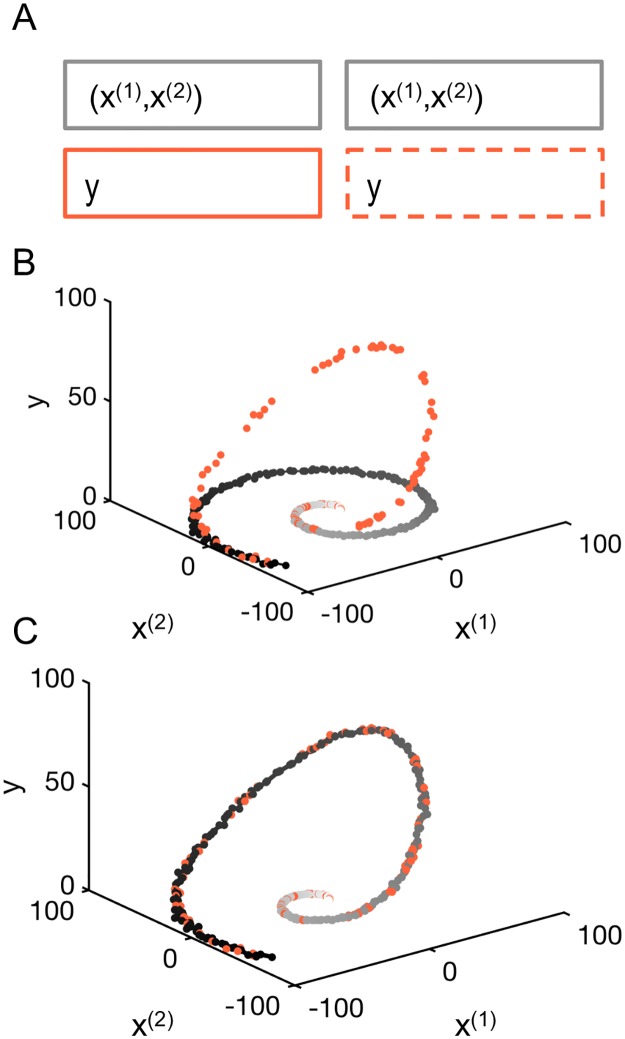
Illustrative example. (A) Matrix formulation of the problem with 120 labeled samples, (((*x*^(1)^(*t*_1_), *x*^(2)^(*t*_1_), *y*(*t*_1_)), …, ((*x*^(1)^(*t*_*l*_), *x*^(2)^(*t*_*l*_), *y*(*t*_*l*_))), and 300 unlabeled samples (((*x*^(1)^(*t*_*l*+1_), *x*^(2)^(*t*_*l*+1_)), *t*_*l*+1_), …, ((*x*^(1)^(*t*_*l*+*u*_), *x*^(2)^(*t*_*l*+*u*_)), *t*_*l*+*u*_)). (B) The points are distributed on a non-linear 1-dimensional manifold in the (*x*^(1)^, *x*^(2)^) - plane. Some points, the snapshots, contain a value for the signal. (C) Result of the interpolation on the nonlinear manifold using the harmonic extension algorithm.

To mimic the setting of data fusion with three modalities, ((*x*^(1)^, *x*^(2)^), *t*, *y*), we consider the following situation: suppose that one acquires a set of labeled points, i.e. a set of *l* triplets, (((*x*^(1)^(*t*_1_), *x*^(2)^(*t*_1_)), *y*(*t*_1_)), …, ((*x*^(1)^(*t*_*l*_), *x*^(2)^(*t*_*l*_)), *y*(*t*_*l*_))) and a set of *u* unlabeled, but timestamped, points, (((*x*^(1)^(*t*_*l*+1_), *x*^(2)^(*t*_*l*+1_)), *t*_*l*+1_), …, ((*x*^(1)^(*t*_*l*+*u*_), *x*^(2)^(*t*_*l*+*u*_)), *t*_*l*+*u*_)), as shown in Fig 3A and 3B. The pairwise similarity measures *w*_*i*,*j*_ are computed using Euclidean norm between pairs of data points (*x*^(1)^(*t*_*i*_), *x*^(2)^(*t*_*i*_)) and (*x*^(1)^(*t*_*j*_), *x*^(2)^(*t*_*j*_)). In this case, there are no outliers, so the standard Euclidean distance is well suited and there is no need to consider other distance measures.

Then, using Eq (2) it is possible to estimate *y* = *f*(*x*) on the set of unlabeled data points using the harmonic extension algorithm. The results are shown in Fig 3C. We then directly obtain *y* as a function of *t* by composition using the known time stamps (*t*_*l*+1_, …, *t*_*l*+*u*_).

The accuracy of the estimated multivariable dynamics can be assessed using a K-fold validation strategy on the labeled samples (S1 Fig and Materials and methods). For the chosen set of parameters and the size of the dataset, the error is ∼ 1%. As expected for the semi-supervised learning framework, the error decreases with the addition of new unlabeled data points. This example demonstrates how the proposed approach successfully recovers multivariable dynamics from heterogeneous datasets that combine continuous views for part of the state variables and snapshots that report several states without direct temporal information.

### Fusion of imaging datasets

As a representative dataset from imaging studies of multivariable dynamics in living systems, we use a collection of ∼ 1000 images each of which reveals the spatial position of the nuclei and either a timestamp or the distribution of one or several components of the DV patterning network (Fig 1D). To apply the semi-supervised learning approach to data fusion to this dataset we need to compute pairwise similarities between the images using the common channel. Prior to this, we took several preprocessing steps that aim to minimize image variability associated to sample handling, microscope calibration and imaging. First, the images were registered to align their ventral-most points. The images were then resized and cropped such that the embryos occupy 80% of the image. All images were resized to 100 by 100 pixels. To overcome local variations of image intensity, we computed a local average using a Gaussian kernel, and then renormalized the image by that value. We also applied a logistic function to the images to handle contrast variability, S2 Fig.

Most importantly, to ensure that pairwise differences between images are insensitive to small translations or deformations, we applied the scattering transform [22] and compared the resulting transform vectors. The scattering transform of an image is a signal representation obtained by alternating wavelet decompositions and pointwise modulus operators. We found that second-order scattering coefficients with an averaging scale of 64 pixels provided sufficient invariance. These are computed using the ScatNet toolbox [23, 24]. The result is a vector of dimension 784 for each image. The point clouds corresponding to each of the 11 datasets were centered separately. It has been shown that the Euclidean distance on the scattering transform is locally invariant to translation and stable to deformation of the original image [22]. For this reason, we compare these 784-dimensional vectors using the Euclidean norm. The corresponding low-dimensional manifold on which the data points lie is shown on S4 Fig.

**Fig 4:**
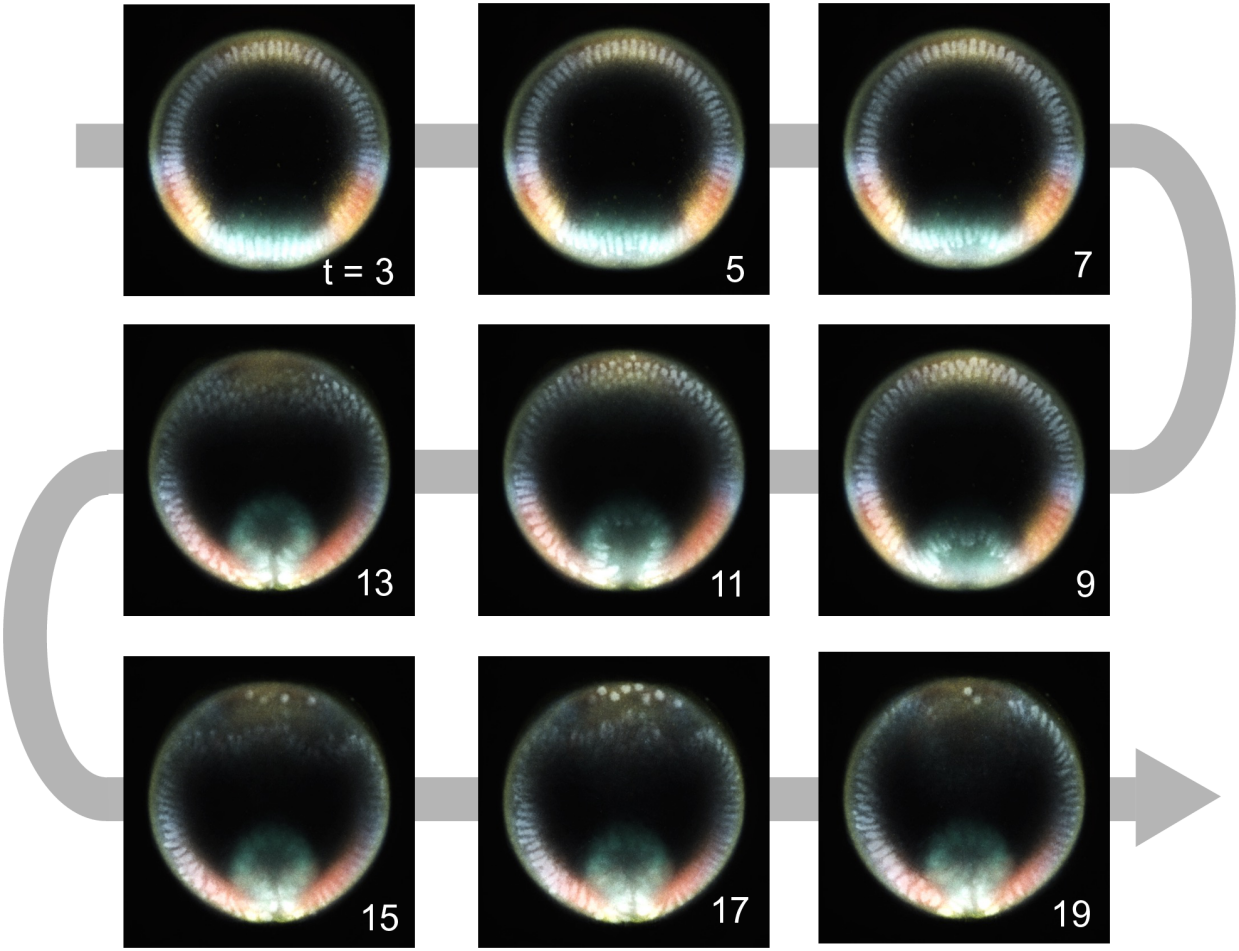
Colored movie frames obtained with our data fusion algorithm. The temporal resolution is 2 min, extracted from a 30 s resolution movie. The time stamps on the images are in min and indicate elapsed time from the start of the live movie. The colors correspond to dpERK (red), Dl (pink), *rho* (yellow), *ind* (blue), Twi (green).

**Fig 5:**
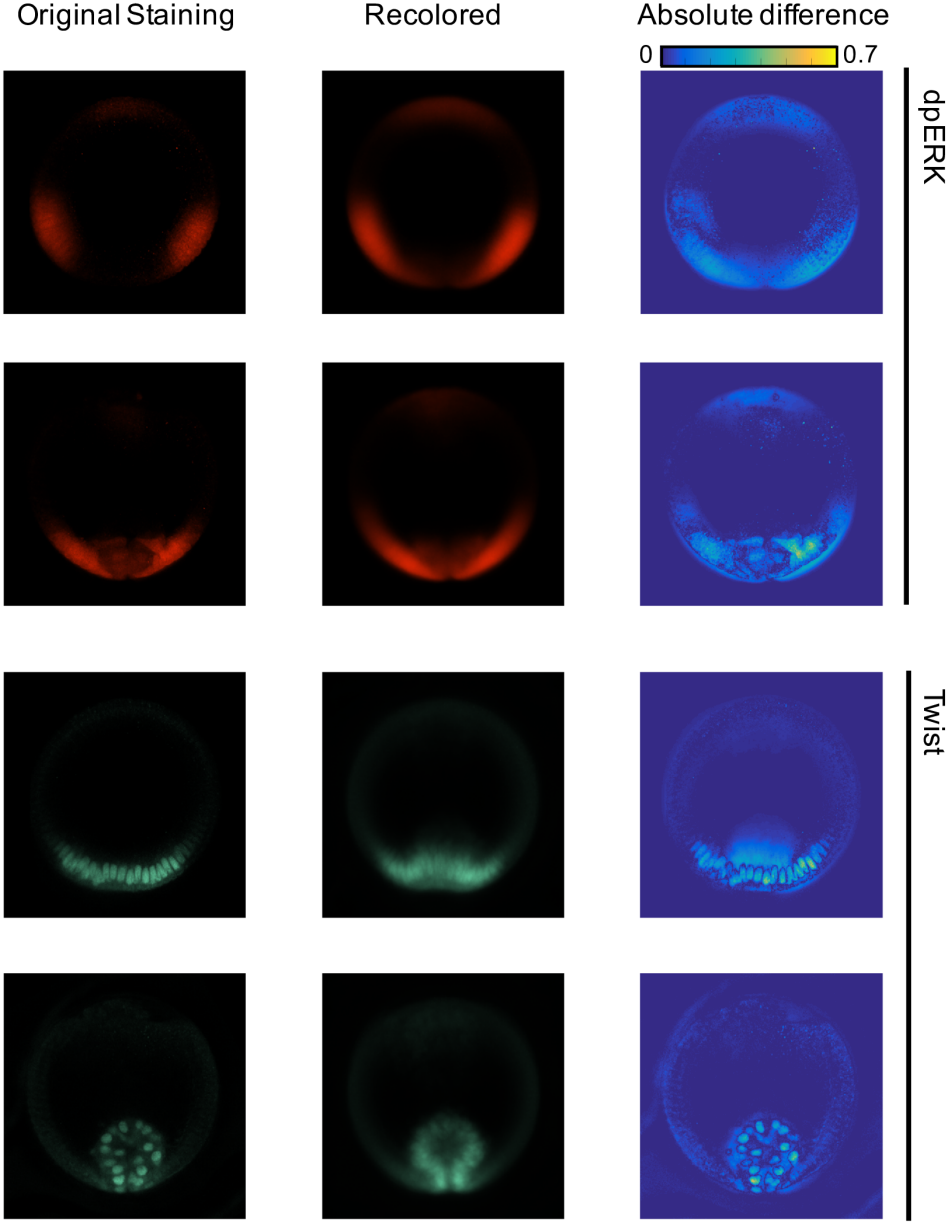
Examples showing how the algorithm performs on four fixed samples. The first column shows the original measurement, the second column shows the result of recoloring the snapshot through K-fold cross validation, and the third column shows the absolute difference between the original and recolored images, normalized by the signal range.

For each of the 512x512 pixels of each live movie frames, there is a common channel reporting the nuclei spatial position and there are 5 channels that we would like to complete. These channels contain the information about the spatial distributions of one enzyme (dpERK), two transcription factors (Twist and Dorsal), and transcripts of two genes (*ind* and *rho*). We thus solved the data fusion problem for each pixel and each channel, leading to 5x512x512 semi-supervised learning solutions. The combination of labeled and unlabeled datasets is described on S2 Table. The result is a multivariable trajectory for the joint dynamics of tissue shape and five molecular components within the regulatory network that patterns the DV axis of the embryo (Fig 4). To evaluate the accuracy of the method, we computed the cross-validation error for each pixel and averaged over the entire images. We found that the normalized absolute error is of 0.9–2.5% of the signal range when considering the various modalities of the entire experimental datasets (S3 Table). We show how the algorithm performs on several examples in Fig 5.

### Discussion

We presented a formal approach to synthesizing developmental trajectories. By posing the task of data fusion as a semi-supervised learning problem, we obtained a closed-form expression for the estimated values of all variables using harmonic extension. The reconstructed trajectories provide the basis for the more advanced mechanistic studies of multivariable processes responsible for the highly reproducible dynamics of developmental pattern formation. Our approach can also be extended using other semi-supervised learning methods [25], if the dimensionality of the intrinsic geometry is greater than one or if there is no unique common channel among all experiments.

Most of the previous attempts to accomplishing this task explored specific features of developmental systems, such as the expression level of a particular gene, and used a discrete number of temporal classes, usually defined in ad hoc way [16, 26]. Our approach reconstructs continuous time dynamics and relies on the intrinsic geometry of multidimensional datasets. Some limitations might appear when considering fluorescent reporters for intrinsically variable processes, and thus not smooth, such as MS2 reporters for nascent transcripts [27]. However, our method is readily applicable to datasets stored in established public databases of gene expression patterns such as the BDGP Resources [28] or the FlyEx database [29] and could serve to animate other pathways such as the segmentation cascade in the early fly development.

We conclude by pointing out two directions for the future extensions and applications of the presented approach. First, while there are no conceptual limitations in using the presented matrix completion framework to studies of pattern formation and morphogenesis problems in three dimensions [30], it is important to increase the computational efficiency of our approach, which can be done at multiple levels, starting with dimensionality reduction at the preprocessing step. At the same time, for a large class of patterning processes that happen on the surfaces of epithelial sheets, one can use the recently developed “tissue cartography” approach to first flatten the three-dimensional images [5], which should make our approach directly applicable. Second, following the step of data fusion, one can attempt to model the observed multivariable dynamics. Here one can employ several modeling methodologies, from mechanistic modeling of specific molecular and tissue-level processes [31–35], to equation-free approaches, which aim to deduce the underlying mechanisms directly from data [36, 37].

## Materials and methods

Extended Materials and Methods are presented in S1 Text.

### Image datasets

All images are cross-sections of *Drosophila* embryos taken at ∼ 90*μ*m from the posterior pole. Time-lapse movies were obtained using a Nikon A1-RS confocal microscope with a 60x Plan-Apo oil objective. The nuclei were stained with Histone-RFP. A total of 7 movies was acquired with a time resolution of 30 seconds per frame. All movies start about 2.5 hr after fertilization and end after about 20 min after gastrulation starts (about 3.3 hr after fertilization). Four datasets of fixed images were acquired to visualize nuclei, protein expression of dpERK, Twist, and Dorsal, and mRNA expression of ind and rho. Immunostaining and fluorescent in situ hybridization protocols were used as described before [16]. DAPI (1:10,000; Vector laboratories) was used to visualize nuclei. Rabbit anti-dpERK (1:100; Cell Signaling), mouse anti-Dorsal (1:100; DSHB), rat anti-Twist (1:1000; gift from Eric Wieschaus, Princeton University), sheep anti-digoxigenin (1:125; Roche), and mouse anti-biotin (1:125; Jackson Immunoresearch) were used as primary antibodies. Alexa Fluor conjugates (1:500; Invitrogen) were used as secondary antibodies. Stained embryos were imaged using Nikon A1-RS confocal microscope with a 60x Plan-Apo oil objective. Embryos were mounted in a microfluidic device for end-on imaging, as described previously [16, 38]. The first dataset contains 108 images stained with rabbit anti-dpERK and rat anti-Twist antibodies. The second dataset contains 59 images stained with mouse anti-Dorsal antibody, rabbit anti-dpERK antibody, and ind-DIG probe. The third dataset contains 58 images stained with ind-biotin probe, rho-DIG probe, and rabbit-dpERK antibody. The fourth dataset contains 30 images stained with rat anti-Twist antibody, ind-biotin probe, and rho-DIG probe. The distribution of the datasets as labeled and unlabeled data depending on the considered variable is summarized on S2 Table. Raw images can be found in Supplementary Files on the public github repository https://github.com/paulvill/data-fusion-images, see S2 Text.

### The affinity matrix

The affinity matrix *W* = (*w*_*i*,*j*_) is computed using a Gaussian kernel $w_{i,j}=\text{exp}\left(-\frac{∥x_{i}-x_{j}{∥}^{2}}{\sigma_{i}\sigma_{j}}\right)$ with scaling parameters *σ*_*i*_ and *σ*_*j*_ computed locally as the average of the distance with respect to the 10 closest neighbors as described in S1 Text. We used the Euclidean norm in the space of scattering transformed data. The resulting affinity matrix is shown on S3 Fig. The corresponding underlying one-dimensional manifold is shown on S4 Fig.

### Computing the cross validation error

The K-fold cross validation error was computed by extracting subsamples of the labeled data points and the semi-supervised learning framework was used to predict the value of the labels on them. For the image datasets, we computed the absolute error between the actual value of pixel intensity to the predicted one. The absolute error was then normalized by the range of the signal computed from the entire set of images for a given channel. The number of bins K was chosen so that the number artificially unlabeled data points was about 20. The results for each dataset are shown in S3 Table and described in S1 Text.

### Movie coloring

The result of data fusion led to multimodal time lapses of developing embryo showing nuclei and the spatio-temporal dynamics of dpERK, Dl, *rho*, *ind*, and Twi. The images were colored using the color code shown in S4 Table, i.e. dpERK (red), Dl (pink), *rho* (yellow), *ind* (blue), Twi (green). A resulting colored movie is provided in Supplementary Files 2.

### Code implementation

The semi-supervised framework used to accomplish the task of data fusion is completely implemented in the open-source MATLAB library and fully runs in GNU Octave. It is available as Supplementary Software on the public github repository https://github.com/paulvill/data-fusion. See S2 Text for a description of the main components of the library.

## Supporting information

S1 Fig K-fold cross validation on the illustrative example. A) Setting with *K* = 5, there are 120 labeled points and the number of unlabeled points varies from 0 to 300. B) The normalized absolute error as a function of the number of unlabeled points. There are 100 repetitions for each number of unlabeled data points.
(TIF)

S2 Fig Illustration of the image preprocessing steps applied on the nuclei channel. The first line shows images resulting from rotation and centering steps. The second line shows images resulting from intensity renormalization. The third line shows images resulting from contrast increase. The first two columns show early and later stages from movie frames stained with Histone-RFP. The last two columns represent early and later stages from fixed samples stained with DAPI.
(TIF)

S3 Fig Affinity matrix *W* = (*w*_*i*,*j*_) obtained by comparing images as described by equation (6) in S1 Text is shown as a heatmap. The white squares identify each of the 11 datasets. The first 7 correspond to live movies, the last 4 correspond to the datasets of fixed images.
(TIF)

S4 Fig Low-dimensional embedding of the 11 datasets obtained by diffusion maps. Each dot is a point and each color is a different dataset. The top left panel shows the points obtained by embedding the points in the first three diffusion map coordinates. The top right panel shows the data points in the plane formed by the first two diffusion map coordinates, while the two bottom panels show the embedding in the planes obtained with the first and third (left) or second and third (right) diffusion map coordinates. Some outliers were filtered out for visualization purposes if their closest neighbor distance was at least twice the median closest neighbor distance, leading to a very well-defined 1-dimensional manifold.
(TIF)

S1 Table Values of the parameters for intensity renormalization and contrast increase for each of the experimental datasets (S1 Text). (PDF)

S2 TableDistribution of the datasets into labeled and unlabeled sets depending on the modality.We refer to Ω(*m*) as the set of labeled datapoints, while $\bar{\Omega (m)}$ is the set of unlabeled data points for the *m*th modality.(PDF)

S3 Table Normalized Absolute Error obtained by K-fold cross-validation for each modality of each dataset. In each case, we performed 10 repetitions, where the labeled samples are distributed randomly among the K bins, and the 309 unlabeled data points are chosen randomly. The error is then averaged over 10 repetitions. More details about the Normalized Absolute Error can be found in S1 Text.
(PDF)

S4 Table Color scheme used to color the final movie. (PDF)

S1 Text Detailed description of the semi-supervised learning framework and its applications to the illustrative example and the experimental datasets. (PDF)

S2 Text Detailed description of the supplementary software and the supplementary files. (PDF)
