# Supplementary figures and images for: Synthesizing developmental trajectories
Source: PLoS Comput Biol. 2017 Sep 18;13(9):e1005742. doi: 10.1371/journal.pcbi.1005742 (PMC5619836; doi:10.1371/journal.pcbi.1005742)

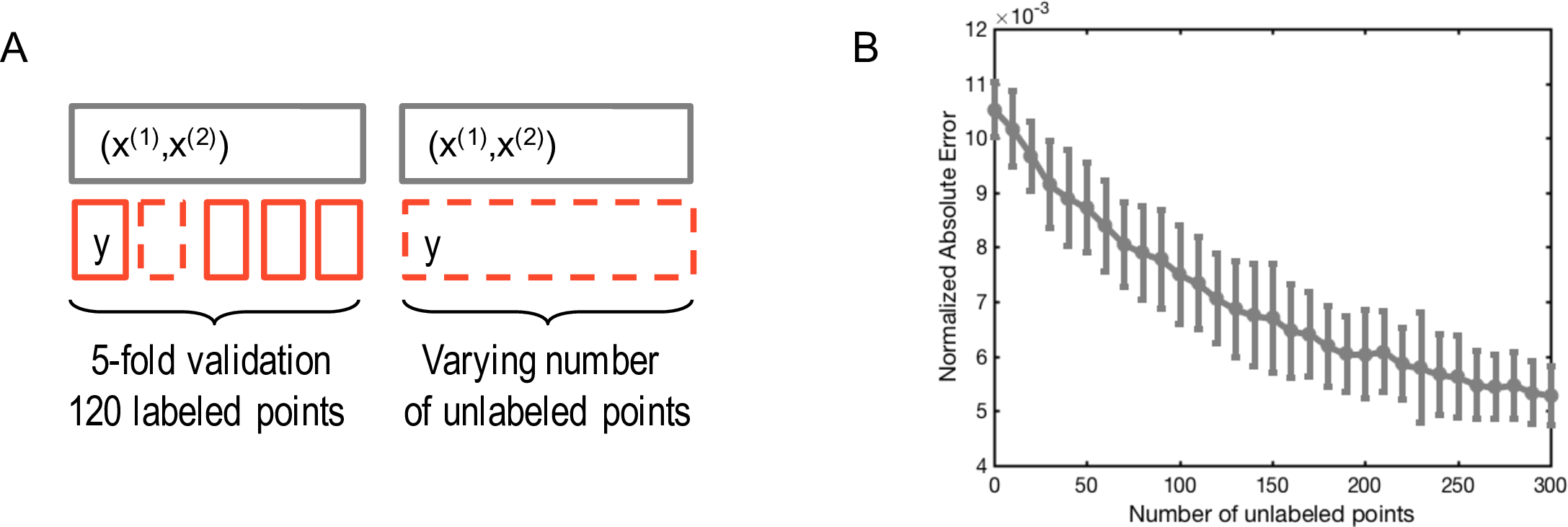

Supplement: S1 Fig — A) Setting with K = 5, there are 120 labeled points and the number of unlabeled points varies from 0 to 300. B) The normalized absolute error as a function of the number of unlabeled points. There are 100 repetitions for each number of unlabeled data points. (TIF) [file pcbi.1005742.s001.tif]

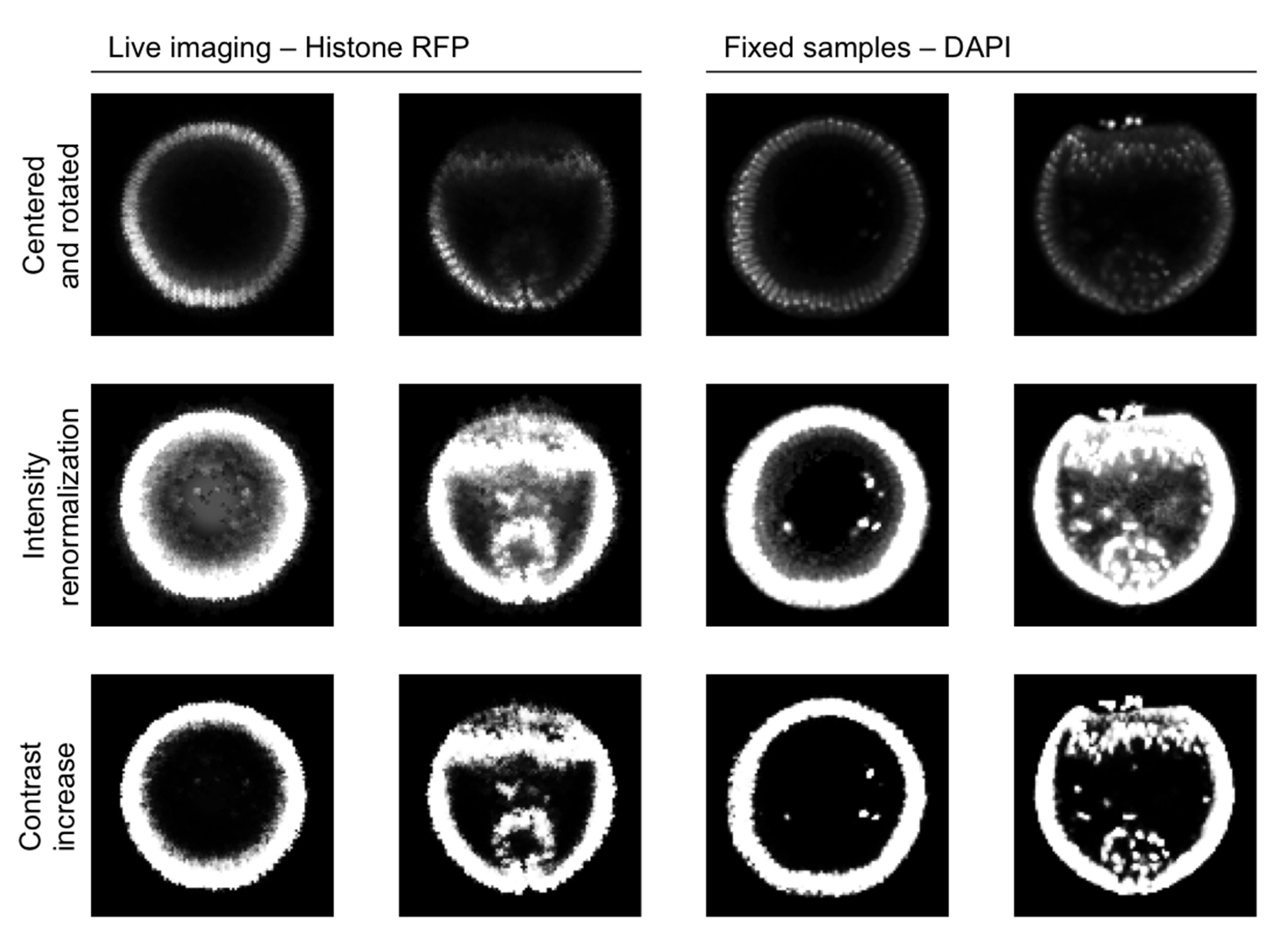

Supplement: S2 Fig — The first line shows images resulting from rotation and centering steps. The second line shows images resulting from intensity renormalization. The third line shows images resulting from contrast increase. The first two columns show early and later stages from movie frames stained with Histone-RFP. The last two columns represent early and later stages from fixed samples stained with DAPI. (TIF) [file pcbi.1005742.s002.tif]

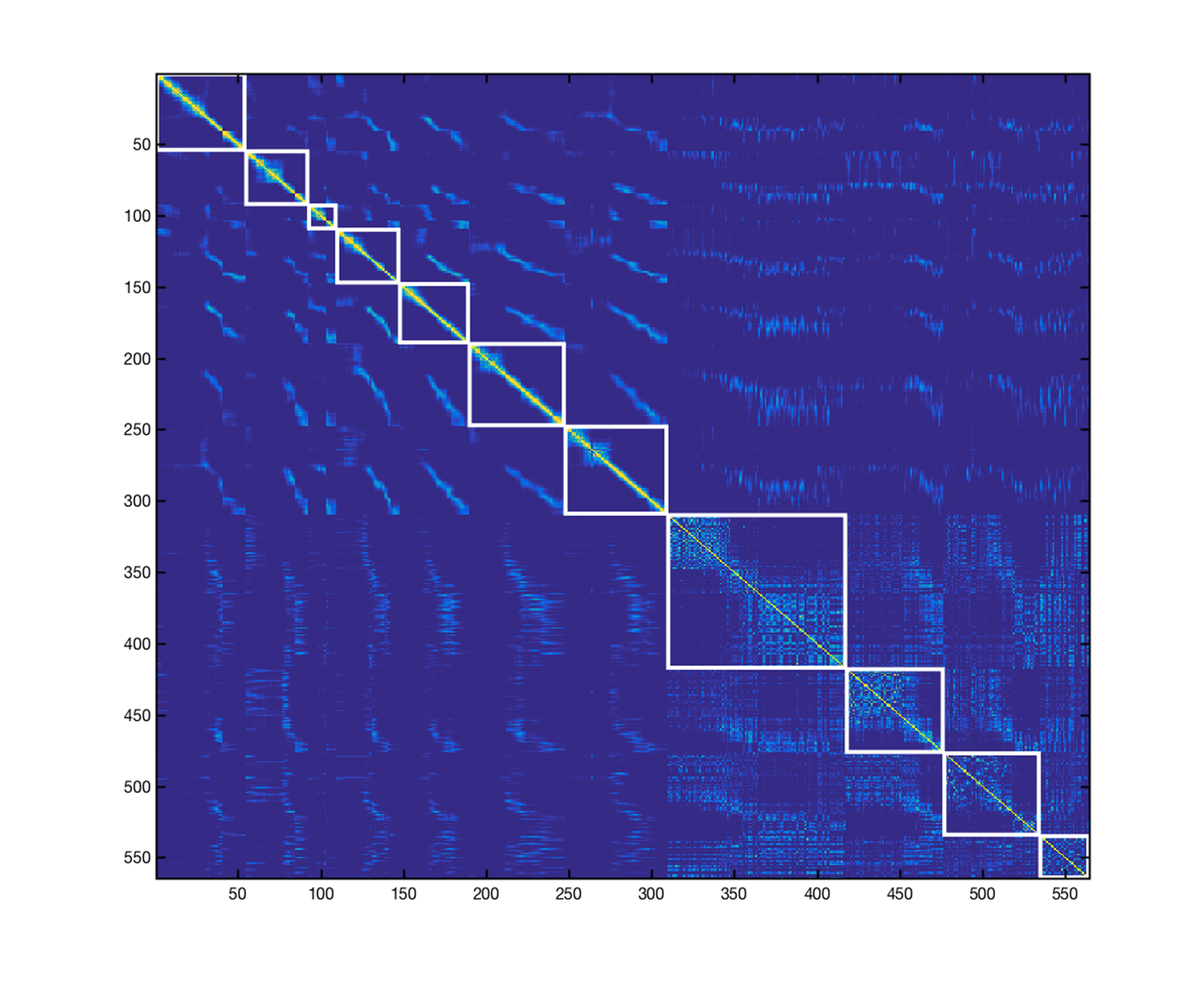

Supplement: S3 Fig — The white squares identify each of the 11 datasets. The first 7 correspond to live movies, the last 4 correspond to the datasets of fixed images. (TIF) [file pcbi.1005742.s003.tif]

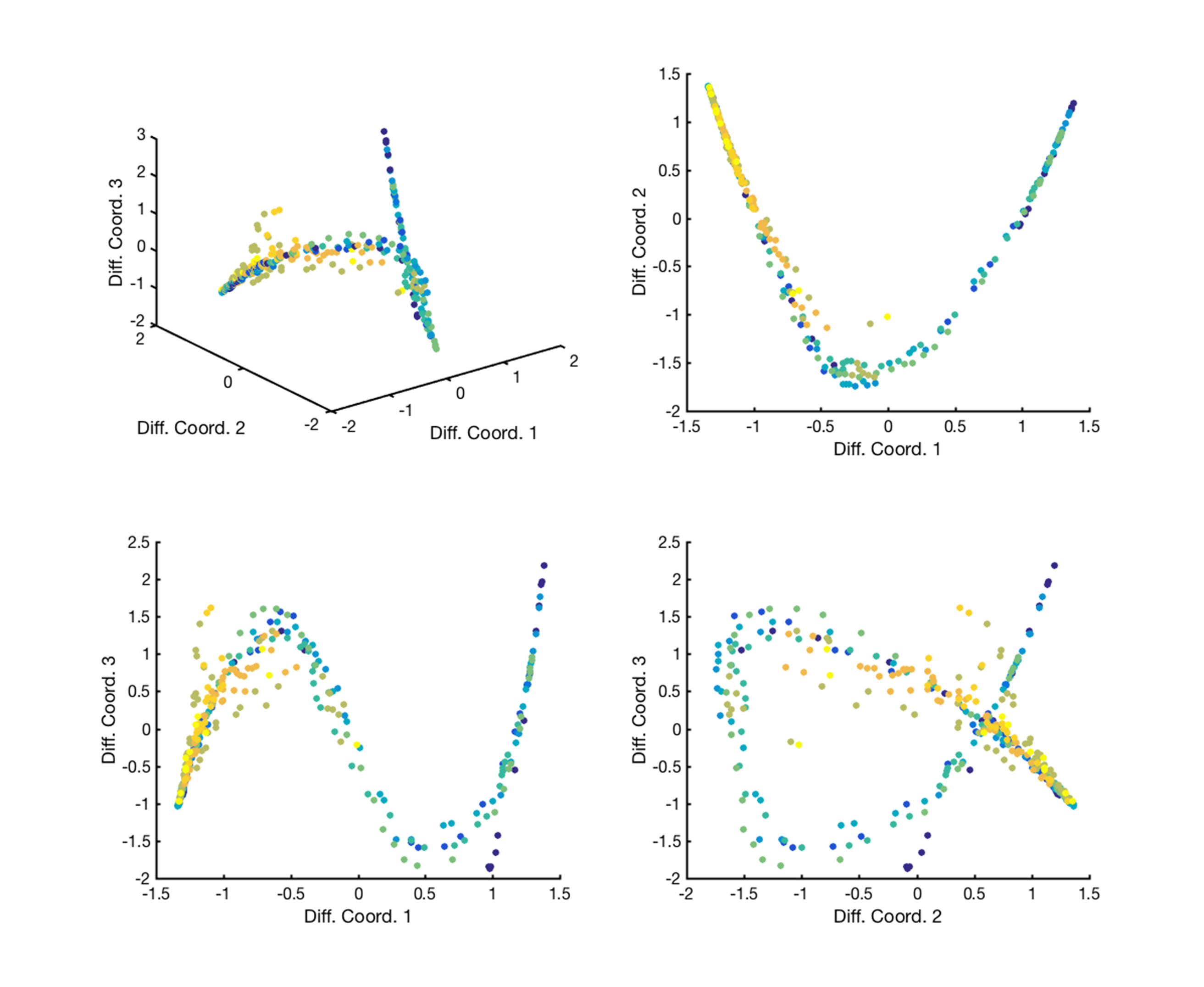

Supplement: S4 Fig — Each dot is a point and each color is a different dataset. The top left panel shows the points obtained by embedding the points in the first three diffusion map coordinates. The top right panel shows the data points in the plane formed by the first two diffusion map coordinates, while the two bottom panels show the embedding in the planes obtained with the first and third (left) or second and third (right) diffusion map coordinates. Some outliers were filtered out for visualization purposes if their closest neighbor distance was at least twice the median closest neighbor distance, leading to a very well-defined 1-dimensional manifold. (TIF) [file pcbi.1005742.s004.tif]
