# Supplementary material for: Synthesizing developmental trajectories
Source: PLoS Comput Biol. 2017 Sep 18;13(9):e1005742. doi: 10.1371/journal.pcbi.1005742 (PMC5619836; doi:10.1371/journal.pcbi.1005742)
Supplement: S1 Table — (PDF) [file pcbi.1005742.s005.pdf]

	Live Movies	Dataset 1	Dataset 2	Dataset 3	Dataset 4
ϵ	0.01	0.01	0.01	0.05	0.01
c	10	10	10	5	10
a	10	10	10	15	10
b	0.8	1.0	1.0	0.5	1.0

Values of the parameters for intensity renormalization and contrast increase for each of the experimental datasets (S1 Text).
