# Supplementary material for: Synthesizing developmental trajectories
Source: PLoS Comput Biol. 2017 Sep 18;13(9):e1005742. doi: 10.1371/journal.pcbi.1005742 (PMC5619836; doi:10.1371/journal.pcbi.1005742)
Supplement: S2 Table — We refer to Ω(m) as the set of labeled datapoints, while Ω(m)¯ is the set of unlabeled data points for the mth modality. (PDF) [file pcbi.1005742.s006.pdf]

	Live Movies	Dataset 1	Dataset 2	Dataset 3	Dataset 4
$m = 1$ dpERK	$\overline{\Omega(1)}$	$\Omega(1)$	$\Omega(1)$	$\Omega(1)$	$\overline{\Omega(1)}$
$m = 2$ Twist	$\overline{\Omega(2)}$	$\Omega(2)$	$\overline{\Omega(2)}$	$\overline{\Omega(2)}$	$\Omega(2)$
$m = 3$ Dorsal	$\overline{\Omega(3)}$	$\overline{\Omega(3)}$	$\Omega(3)$	$\overline{\Omega(3)}$	$\overline{\Omega(3)}$
$m = 4$ ind	$\overline{\Omega(4)}$	$\overline{\Omega(4)}$	$\Omega(4)$	$\Omega(4)$	$\Omega(4)$
$m = 5$ rho	$\overline{\Omega(5)}$	$\overline{\Omega(5)}$	$\overline{\Omega(5)}$	$\Omega(5)$	$\Omega(5)$

Distribution of the datasets into labeled and unlabeled sets depending on the modality. We refer to $\Omega(m)$ as the set of labeled datapoints, while $\overline{\Omega(m)}$ is the set of unlabeled data points for the mth modality.
