# Supplementary material for: Synthesizing developmental trajectories
Source: PLoS Comput Biol. 2017 Sep 18;13(9):e1005742. doi: 10.1371/journal.pcbi.1005742 (PMC5619836; doi:10.1371/journal.pcbi.1005742)
Supplement: S3 Table — In each case, we performed 10 repetitions, where the labeled samples are distributed randomly among the K bins, and the 309 unlabeled data points are chosen randomly. The error is then averaged over 10 repetitions. More details about the Normalized Absolute Error can be found in S1 Text. (PDF) [file pcbi.1005742.s007.pdf]

	Normalized Absolute Error (in %) 309 unlabeled data points
Dataset 1	
dpERK	2.37
Twist	2.03
Dataset 2	
dpERK	2.01
Dorsal	1.10
ind	1.45
Dataset 3	
dpERK	1.57
ind	0.9
rho	1.11
Dataset 4	
Twist	2.18
ind	1.63
rho	1.84

Normalized Absolute Error obtained by K-fold cross-validation for each modality of each dataset. In each case, we performed 10 repetitions, where the labeled samples are distributed randomly among the K bins, and the 309 unlabeled data points are chosen randomly. The error is then averaged over 10 repetitions. More details about the Normalized Absolute Error can be found in S1 Text.
