# Supplementary material for: Synthesizing developmental trajectories
Source: PLoS Comput Biol. 2017 Sep 18;13(9):e1005742. doi: 10.1371/journal.pcbi.1005742 (PMC5619836; doi:10.1371/journal.pcbi.1005742)
Supplement: S4 Table — (PDF) [file pcbi.1005742.s008.pdf]

	R	G	B
dpERK	213	36	2
Twist	92	188	146
Dorsal	229	195	207
ind	103	139	191
rho	225	223	75

Color scheme used to color the final movie.
