# Supplementary material for: Synthesizing developmental trajectories
Source: PLoS Comput Biol. 2017 Sep 18;13(9):e1005742. doi: 10.1371/journal.pcbi.1005742 (PMC5619836; doi:10.1371/journal.pcbi.1005742)
Supplement: S1 Text — (PDF) [file pcbi.1005742.s009.pdf]

1. Semi-supervised learning

Problem Setting. We define the problem of data fusion using semi-supervised learning in general terms. A classical setting is to have a modality x belonging to some space $\mathcal{X}$ which is common to all experiments and M other modalities $(y^{(1)}, \dots, y^{(M)})$ that vary across experiments. The difficulty here is that only a certain number of modalities can be measured simultaneously, so our goal is to fill in the missing modalities. The critical assumption that we use is that the common modality x is sufficient to determine the other modalities. In other words, there exists a set of functions $f_*^{(1)}, \dots, f_*^{(M)}$ such that $y^{(m)} = f_*^{(m)}(x)$ for all $1 \leq m \leq M$ and all x.

Let us represent the ith experiment, here containing the common modality x and the modalities m_1, and m_2 as an example by

$$\begin{pmatrix} x_i \\ \times \\ \vdots \\ \times \\ y_i^{(m_1)} \\ \times \\ \vdots \\ \times \\ y_i^{(m_2)} \\ \times \\ \vdots \\ \times \end{pmatrix},$$

where $\times$ denotes an empty, or unmeasured, modality. The corresponding completed vector would then be

$$\begin{pmatrix} x_i \\ f^{(1)}(x_i) \\ \vdots \\ f^{(M)}(x_i) \end{pmatrix}$$

where $f^{(1)}, \dots, f^{(M)}$ are our estimates of the underlying functions $f_*^{(1)}, \dots f_*^{(M)}$ with $f^{(m_1)}(x_i) = y_i^{(m_1)}$ and $f^{(m_2)}(x_i) = y_i^{(m_2)}$.

Problem formulation: The problem of fusing multiple modalities can be summarized as completing the following matrix using the information on the row x while preserving the continuity of the underlying manifold.

$$\begin{pmatrix} x_1 & \dots & x_n \\ y_1^{(1)} & \dots & y_n^{(1)} \\ & \vdots & \\ y_1^{(M)} & \dots & y_n^{(M)} \end{pmatrix}.$$

For the mth modality, we denote the set of experiments where it is measured by $\Omega(m)$. This is a subset of $\{1, \dots, n\}$, in which it has the complement $\overline{\Omega(m)} = \{1, \dots, n\} \backslash \Omega(m)$. We shall refer to $\Omega(m)$ as the set of labeled datapoints, while $\overline{\Omega(m)}$ is the set of unlabeled data points for the mth modality. Note that we do not impose any particular structure on the sets of labeled points $\Omega(m)$. They can occur in any order and vary in size between the different modalities.

Multiple semi-supervised learning by harmonic extension The problem of completing the mth row of the matrix using the measured elements $Y^{(m)} = \{y_i^{(m)} : i \in \Omega(m)\}$ can be posed as a semi-supervised learning problem, which consists in learning the mapping $f^{(m)}(x) = y^{(m)}$ on each of the points $(x_1, ..., x_n)$ (1). To simplify the notation, we shall consider the n values of $f^{(m)}$ on these points as a column vector in $\mathbb{R}^n$. In addition, we denote the subset of its values when restricted to $\Omega(m)$ and $\overline{\Omega(m)}$ by $f^{(m)}|_{\Omega(m)} \in \mathbb{R}^{|\Omega(m)|}$ and $f^{(m)}|_{\overline{\Omega(m)}} \in \mathbb{R}^{|\overline{\Omega(m)}|}$, respectively, where $|A|$ denotes the number of elements in the set A. Since $Y^{(m)}$ is also a column vector in $\mathbb{R}^{|\Omega(m)|}$, we formulate our problem as

$$f^{(m)} = \underset{\substack{f \in \mathbb{R}^n \\ f|_{\Omega(m)} = Y^{(m)}}}{\operatorname{argmin}} \; \frac{1}{2} \sum_{i=1}^{n} \sum_{j=1}^{n} w_{i,j} \| f(x_i) - f(x_j) \|^2 \qquad [1]$$

where $w_{i,j}$ is a measure of the affinity between x_i and x_j that varies between 0 and 1, we compute it in 6.

This problem can be rewritten in terms of a matrix for each row (modality) m. First we introduce the Laplacian matrix L, given by

$$L = D - W$$

where $D = \operatorname{diag}(d_1, \dots, d_n)$ is the diagonal matrix with entries $d_1, \dots, d_n$ and $d_i = \sum_{j=1}^{n} w_{i,j}$ and $W = (w_{i,j})_{1 \le i,j \le n}$. With this, our optimization problem can be rewritten as

$$f^{(m)} = \underset{\substack{f \in \mathbb{R}^n \\ f|_{\Omega(m)} = Y^{(m)}}}{\operatorname{argmin}} \; f^{\mathrm{T}} L f. \qquad [2]$$

This is a quadratic optimization problem with linear constraints and can be solved by differentiating and setting the derivative to zero. The solution $f^{(m)}$, on the subsets $\Omega(m)$ and $\overline{\Omega(m)}$, is given by

$$\begin{aligned} f^{(m)}|_{\Omega(m)} &= Y^{(m)} \\ f^{(m)}|_{\overline{\Omega(m)}} &= \left(D|_{\overline{\Omega(m)},\overline{\Omega(m)}} - W|_{\overline{\Omega(m)},\overline{\Omega(m)}} \right)^{-1} W|_{\overline{\Omega(m)},\Omega(m)} Y^{(m)} \end{aligned} , \qquad [3]$$

where we have adopted the notation of $M|_{A,B}$ to denote the submatrix of M obtained by extracting the rows in the index set A and the columns in index set B.

The solution Eq. (3) can be rewritten as

$$f^{(m)}(i) = Y^{(m)}(i) \quad \forall i \in \Omega(m) \qquad [4]$$

and

$$f^{(m)}(i) = \frac{1}{D(i,i)} \sum_{j=1}^{n} w_{i,j} f^{(m)}(j) \quad \forall i \in \overline{\Omega(m)}. \qquad [5]$$

Note that Eq. (5) is an implicit equation satisfied by the values. To calculate the desired values of $f^{(m)}$, Eq. (3) must be used.

The above calculations are performed separately for each modality $m = 1, \dots, M$, each solution completing a different row in the overall measurement matrix.

Computing the Affinity Matrix. Given a set of point $(x_1, ..., x_n)$, we compute the affinity matrix $W = (w_{i,j})$ with

$$w_{i,j} = \exp\left(-\frac{\|x_i - x_j\|^2}{\sigma_i \sigma_j} \right) \qquad [6]$$

where the scaling parameters σ_i and σ_j are defined as $\sigma_t = \frac{1}{|\mathcal{N}(x_t)|}\sum_{k\in\mathcal{N}(x_t)} \|x_t - x_k\|$ for every $t = 1, \ldots, n$, where $\mathcal{N}(x_t)$ represents the neighborhood composed of the $|\mathcal{N}(x_t)| = 10$ closest points of x_t (2). The norms here are Euclidean norms (3). This gives us a proximity scale that varies across the manifold if the sampling of the points is not uniform.

K-Fold Cross Validation. To evaluate the accuracy of the reconstruction obtained by data fusion we employ K-fold cross validation (4). This approach estimates the expected prediction error. The idea is to predict the value of labels on a subset of the labeled data points and compare the prediction to the known labels.

Let us consider labeled data points $x|_{\Omega(m)}$ with labels $Y^{(m)}$ and unlabeled data points $x|_{\overline{\Omega(m)}}$. The labeled data points can be uniformly assigned to K bins randomly using an indexing function $\kappa : \Omega(m) \to \{1, ..., K\}$. For each value k of κ, we thus have the index set $\kappa^{-1}(k) = \{i \in \Omega(m) : \kappa(i) = k\}$, a subset of $\Omega(m)$. We can thus define reduced set of labeled measurements $\Omega_k(m) = \Omega(m)\backslash\kappa^{-1}(k)$, with the corresponding set of unlabeled measurements $\overline{\Omega_k(m)} = \overline{\Omega(m)} \cup \kappa^{-1}(k)$.

For $i \in \Omega(m)$, we denote by $f^{(m),-\kappa(i)}(i)$ the label prediction on data point i where $\Omega_{\kappa(i)}(m)$ and $\overline{\Omega_{\kappa(i)}(m)}$ are used as the set of labeled and unlabeled points, respectively. Since $y_i^{(m)}$ is known, we can evaluate the performance of our method by comparing the prediction $f^{(m),-\kappa(i)}(i)$ to the known value $y_i^{(m)}$. Repeating this for all $i \in \Omega(m)$, we calculate the average absolute error normalized by the range for each modality m

$$\mathrm{Err}^{(m)}(f) = \left(\frac{1}{|\Omega(m)|}\sum_{i\in\Omega(m)} |f^{(m),-\kappa(i)}(i) - y_i^{(m)}|\right) / \left(\max(Y^{(m)}) - \min(Y^{(m)})\right). \qquad [7]$$

2. Illustrative example

To demonstrate the efficiency of this technique as an interpolation method on a non-linear manifold, we first consider a toy example with a non-linear 1-dimensional trajectory embedded in 3-dimensional space. Specifically, we have

$$\begin{cases} x^{(1)}(t) & = at\left(\cos(bt) + \epsilon^{(1)}\right) \\ x^{(2)}(t) & = at\left(\sin(bt) + \epsilon^{(2)}\right) \\ y(t) & = ct\exp(-d(t-e)^2) \end{cases} \qquad [8]$$

where, a, b, c, d, e are constants, $\epsilon^{(1)}$ and $\epsilon^{(2)}$ are Gaussian noise sources and t is a real-valued parameter. The set of points $(x^{(1)}(t), x^{(2)}(t))$ forms a 1-dimensional non-linear manifold embedded in the 2-dimensional space as it is parameterized by t. These points correspond to the embryo morphology in our toy example. In the absence of noise, this mapping from t to the 2D plane can be inverted as $t = \frac{1}{|a|}\sqrt{(x^{(1)})^2(t) + (x^{(2)})^2(t)}$. The signal $y(t)$ is a smooth function of t and is thus a smooth function of $(x^{(1)}(t), x^{(2)}(t))$ by composition. In this example, y corresponds to the target modality that we would like to estimate.

To reproduce the setting of data fusion with three modalities, $((x^{(1)}, x^{(2)}), t, y)$, we consider the following situation. Suppose that one can acquire a set of labeled points, i.e. a set of l triplets, $((x^{(1)}(t_1), x^{(2)}(t_1)), y(t_1)), ..., ((x^{(1)}(t_l), x^{(2)}(t_l)), y(t_l))$ and a set of u unlabeled, but timestamped, points, $((x^{(1)}(t_{l+1}), x^{(2)}(t_{l+1})), t_{l+1}), ..., ((x^{(1)}(t_{l+u}), x^{(2)}(t_{l+u})), t_{l+u})$, as shown in Fig. 1A and B. We therefore have $\Omega = \{1, \ldots, l\}$ and $\overline{\Omega} = \{l+1, \ldots, l+u\}$ and $n = l + u$ (since we only consider y as labels, and hence have only one modality to fill, we have suppressed the superscript (m)). The pairwise similarity measures $w_{i,j}$ are computed using Euclidean norm between pairs of data points $(x^{(1)}(t_i), x^{(2)}(t_i))$ and $(x^{(1)}(t_j), x^{(2)}(t_j))$ as presented in 6. Then using the framework presented above, in particular equations Eq. (4) and Eq. (5), it is possible to estimate $y = f(x)$ on the set of unlabeled data points using the harmonic extension algorithm. The results are shown in Fig. 1C. We then directly obtain y as a function of t by composition using the known time stamps $(t_{l+1}, ..., t_{l+u})$.

For this example, we now quantify the evolution of the error as a function of the number of unlabeled samples. The constants used were $a = 1$, $b = 0.1$, $c = 1$, $d = 0.005$, $e = 65$, $l = 120$, and $u = 300$ while t varies from 1 to 100 and $\epsilon^{(1)}$ and $\epsilon^{(2)}$ are sampled according to centered Gaussian distributions with standard deviation 2.02. Using a K-fold validation strategy on the labeled samples, we characterize the accuracy of the reconstruction using the normalized absolute error as described by equation Eq. (7) (S1 Fig A). The results are shown on S1 Fig B. We found that with no additional data points, the absolute error was on average 1.05% of the signal range, and decreased uniformly to 0.53% when considering the problem with an additional 300 unlabeled data points.

3. Experimental Data Sets

Data Sets description. To explore the capabilities of our data fusion strategy, we considered 11 different datasets, each of which consisting of a set of images, obtained either as live movies or fixed samples, and containing a channel recording the spatial distribution of nuclei.

Live Movies. Nikon A1-RS confocal microscope with a 60x Plan-Apo oil objective was used to obtain time-lapse movies of Drosophila embryonic development. Live imaging of Histone-RFP embryos was used to visualize nuclei. A total of 7 movies were obtained, with a time resolution of 30 seconds per frame. All the movies start during nuclear cycle 14 (about 2.5 hr after fertilization) and end after about 20 min after gastrulation starts (about 3.3 hr after fertilization). The number of frames for each movie is 156, 67, 53, 55, 50, 86, and 90.

Fixed samples. In our experiments, 4 datasets were acquired to visualize nuclei, protein expression of dpERK, Twist, and Dorsal, and mRNA expression of ind and rho. Immunostaining and fluorescent in situ hybridization protocols were used as described before (5). DAPI (1:10,000; Vector laboratories) was used to visualize nuclei. Rabbit anti-dpERK (1:100; Cell Signaling), mouse anti-Dorsal (1:100; DSHB), rat anti-Twist (1:1000; gift from Eric Wieschaus, Princeton University), sheep anti-digoxigenin (1:125; Roche), and mouse anti-biotin (1:125; Jackson Immunoresearch) were used as primary antibodies. Alexa Fluor conjugates (1:500; Invitrogen) were used as secondary antibodies. Stained embryos were imaged using Nikon A1-RS confocal microscope with a 60x Plan-Apo oil objective. Embryos were mounted in a microfluidic device for end-on imaging, as described previously (5, 6). All the images were taken at $\sim$90 μm from the posterior pole of the embryo.

Dataset 1 Stained with rabbit anti-dpERK and rat anti-Twist antibodies. Number of samples: 108.

Dataset 2 Stained with mouse anti-Dorsal antibody, rabbit anti-dpERK antibody, and ind-DIG probe. Number of samples: 59.

Dataset 3 Stained with ind-biotin probe, rho-DIG probe, and rabbit-dpERK antibody. Number of samples: 58.

Dataset 4 Stained with rat anti-Twist antibody, ind-biotin probe, and rho-DIG probe. Number of samples: 30.

Preprocessing Steps. In order to compare the images of morphology (nuclei channel) among all the datasets, we used a certain number of preprocessing steps to overcome measurement-dependent variability.

Yolk removal. Unwanted signals in the yolk coming from artifacts in the acquisition process were removed in some images.

Image Rotation. Images were oriented so that the highest Dorsal signal is located at the ventral-most point. Some late-stages embryos were oriented manually.

Image Resizing. The images were resized and cropped such that the embryo would occupy 80% of the image. This was achieved by thresholding the images and computing their bounding box, then resizing and translating the image so that the bounding box occupied the square stretching from 10% to 90% of the image width, both horizontally and vertically. The images were resized to 100 by 100 pixels.

Intensity Renormalization. Since there is some local variation of image intensity that is due to the device used to acquire data and the configuration of the microscope, we wish to remove this from the images before comparing them. This is done by computing a local average using a Gaussian kernel, and then renormalizing the image by that value. Specifically, given an image x, we calculate its local average $y(u) = x \star \phi(u)$, where $\phi(u) = \mathrm{e}^{-u^2/2c^2}$ for some kernel width $c > 0$ and $\star$ denotes convolution. Then the renormalized image is given, for each pixel u, by

$$\frac{y(u)}{y(u)^2 + \epsilon^2} x(u), \qquad [9]$$

where $\epsilon > 0$ is some threshold that prevents the renormalization from exploding when the image is locally close to zero.

Contrast Increase. Another consequence of the experimental setup and the configuration of the microscope is that the contrast differs between measurements. As a result, the range of pixel intensities is not the same for different images of embryos at the same developmental stage. To fix this, we pass the pixel intensities through a logistic function with two parameters. Given an input image x, it is adjusted to give, for each pixel u,

$$\frac{1}{1 + \mathrm{e}^{-a(x(u)-b)}}. \qquad [10]$$

Scattering Transform. To compare images without being sensitive to small translations or deformations, we applied the scattering transform (7) and compared the resulting transform vectors. Differences between images in the scattering space are meant to reflect changes in the underlying morphology as opposed to variation due to the imaging method.

The scattering transform of an image is a signal representation obtained by alternating wavelet decompositions and pointwise modulus operators. The resulting coefficients are locally invariant to translations and stable to deformations. The similarity of the signals can then be measured by computing the Euclidean distance of their scattering transforms. These transforms have enjoyed significant success in classification of images (8, 9), but also time series (10), since these tasks often requires insensitivity to translation and deformation.

For our embryo images, we have found that second-order scattering coefficients with an averaging scale of 64 pixels allow for the proper amount of invariance. These are computed using the ScatNet toolbox (11).

The result is a vector of dimension 784 for each image. The point clouds corresponding to each of the 11 datasets were centered separately.

Computing the affinity matrix. The affinity matrix was computed according to the definition of the Gaussian kernel with an adaptive scaling factor, as described in 6. The result is shown on S3 Fig.

Outlier filtering. Even after applying all the previous steps of image preprocessing some data points were considerably away from the main manifold. First we calculated the closest neighbor of each point. We then kept the points whose closest neighbor distance was less than 3 times the median minimal neighbor distance.

Results. To fuse the channels of the various datasets described in 3, we applied the harmonic extension algorithm described in 1. The data points are preprocessed images of nuclei spatial distribution using the steps presented in 3 to 3 with parameter values as shown in S1 Table and the modalities correspond to the images obtained from the various fluorescent reporters described in section 3 of the SI appendix. The corresponding low-dimensional manifold on which the data points x_i lie is shown on S4 Fig.

We denote by x_i the preprocessed image i of the nuclei channel, it is an element of $\mathbb{R}^{784}$ as the result of scattering transformation applied to a 100×100 image. We denote by $y_i^{(m)}$ the image corresponding to modality m associated to x_i and by $y_i^{(m)}(p)$ its restriction to the pixel p, $y_i^{(m)}(p)$ is an element of $\mathbb{R}$.

For each of the 10000 pixels in the live movie, there are 5 modalities that we would like to fuse: dpERK ($m = 1$), Twist ($m = 2$), Dorsal ($m = 3$), ind ($m = 4$), rho ($m = 5$). We therefore solve the data fusion problem for each pixel and each modality, leading to 50000 semi-supervised learning solutions. The combination of labeled and unlabeled datasets is described on S2 Table.

To evaluate the accuracy of the method, we computed the cross-validation error as described by equation Eq. (7) for each pixel and averaged over the entire images. The range of the signal is

calculated based on the entire images. S3 Table shows the results for each of the datasets where the number of unlabeled data points is 309 (the number of live movie frames), the unlabeled data points are chosen randomly among the live movie frames and the fixed images from the 4 datasets which serve as unlabeled data points. For each of the datasets, we chose K such the number of points within each bin was about 20. We have $K = 6, 3, 3, 2$ for datasets 1 to 4 respectively.

Coloring movies. The resulting fused dataset led to the construction of a multimodal movie, where a different color was attributed to each modality and the RGB values were added in the resulting movie. We used the set of colors presented on S4 Table.

References

1. Zhu X, Ghahramani Z, Lafferty JD (2003) Semi-supervised learning using gaussian fields and harmonic functions in *Proceedings of the 20th International conference on Machine learning (ICML-03)*. pp. 912–919.
2. Zelnik-Manor L, Perona P (2004) Self-tuning spectral clustering. *Advances in neural information processing systems* 17(1601-1608):16.
3. Lederman RR, Talmon R (2014) Common manifold learning using alternating-diffusion. *Yale University, New Haven, CT, USA, Tech. Rep. YALEU/DCS/TR-1497*.
4. Friedman J, Hastie T, Tibshirani R (2001) *The elements of statistical learning*. (Springer series in statistics Springer, Berlin) Vol. 1.
5. Lim B, et al. (2015) Dynamics of inductive erk signaling in the drosophila embryo. *Current Biology* 25(13):1784–1790.
6. Levario TJ, Zhan M, Lim B, Shvartsman SY, Lu H (2013) Microfluidic trap array for massively parallel imaging of drosophila embryos. *Nature protocols* 8(4):721–736.
7. Mallat S (2012) Group invariant scattering. *Comm. Pure Appl. Math.* 65(10):1331–1398.
8. Bruna J, Mallat S (2013) Invariant scattering convolution networks. *IEEE Trans. Pattern Anal. Mach. Intell.* 35(8):1872–1886.
9. Sifre L, Mallat S (2013) Rotation, scaling and deformation invariant scattering for texture discrimination in *IEEE Conf. on Comput. Vis. and Pattern Recognit.* (IEEE), pp. 1233–1240.
10. Andén J, Mallat S (2014) Deep scattering spectrum. *IEEE Trans. Sig. Proc.* 62:4114–4128.
11. Andén J, et al. (2014) Scatnet. *Computer Software. Available: http://www.di.ens.fr/data/software/scatnet/*.
