# Supplementary material for: Synthesizing developmental trajectories
Source: PLoS Comput Biol. 2017 Sep 18;13(9):e1005742. doi: 10.1371/journal.pcbi.1005742 (PMC5619836; doi:10.1371/journal.pcbi.1005742)
Supplement: S2 Text — (PDF) [file pcbi.1005742.s010.pdf]

Supplementary Files 1: This compressed folder contains all the raw images organized into folders: movie1-7, and data_set1-4. The folders movie1-7 correspond to the live movies, while the folders data_set1-4 correspond to stained fixed images. The description of the datasets can be found within each folder or in S1 Text. The files can be accessed at https://github.com/paulvill/data-fusion-images.

Supplementary Files 2: Colored movie. The compressed folder contains frames of a colored movie, with, in the main folder, all the channels combined, nuclei (gray), dpERK (red), Dl (pink), rho (yellow), ind (blue), Twi (green), using the color map presented in S4 Table. The number of each image corresponds to a time step, and they have been extracted from movie 5. The subfolder "grayscale" contains the actual value of the variables in separate frames, the name of each image indicates the variable considered, while the number of each image corresponds to the time step. The files can be accessed at https://github.com/paulvill/data-fusion-output.

Supplementary Software: The compressed folder contains the MATLAB library implementing the data fusion algorithm on an illustrative example and on experimental cross-sections of developing fly embryos. The files can be accessed at https://github.com/paulvill/data-fusion. The file README.md contains instructions on how to run the code. The main scripts are found in the examples and experiments folders and consist of:

- spiral_example.m: implements the data fusion algorithm on a non-linear 1-dimensional trajectory in a 3-dimensional space, nicknamed "spiral".
- spiral_example_cross_validation.m: implements the K-fold cross-validation on the "spiral".
- experimental_dataset.m: implements the data fusion on the experimental datasets leading to a multimodal movie containing the spatio-temporal dynamics of 5 chemical species on top of morphological changes.
- experimental_dataset_cross_validation.m: implements the K-fold cross validation on the experimental datasets.

When working on the spiral example, the structure of the code can be decomposed into the following steps:

1. Simulate the data
   - The simulation is performed directly by implementing the system of equations (8) in S1 Text.
2. Pairwise differences and affinity matrix
   - `d = distances(vecs);`
     This function calculates the euclidean distance between vectors vecs.
   - `[A,eps]=AffinityFromDistance( d, numNeighbors)`
     This function computes an affinity matrix from given distances d. $A_{ij} = \exp(d_{ij}^2/(\sqrt{\epsilon_i}\sqrt{\epsilon_j})$, where ϵ_i a scaling constant around the i-th point. ϵ_i is taken to be the average of the square of the distances from the i-th point to its numNeighbors nearest neighbors.
3. Harmonic extension

- `[inv_u] = ssl_estimate(W, indl, indu)`

  This function computes the matrix required to obtain the predicted sample values on unlabeled data points from labeled data points. W is the affinity matrix containing the pairwise difference $w_{i,j}$ between all the data points. The output of this function is the matrix inv_u, which is necessary to compute the predicted labels from the unlabeled ones, f_u = inv_u*Y where Y correspond to the labels.

When working on the experimental datasets, the structure of the code can be decomposed into the following steps which include additional preprocessing steps to the artificial toy example:

1. Load datasets

   - `movies = load_movie_set(movie_opt);`

     This function takes as input an options structure specifying the location and format of the movie or snapshots files and returns a structure containing the loaded movies or snapshots.

2. Preprocessing steps

   - `IMAGE2 = mean_center_image(IMAGE, CHANNEL, RESIZE_IMAGE, edge_tol)`

     This function translates an image so that it is (approximately) mean-centered in the frame. It is used for individual images.

   - `IMAGE_STACK2 = mean_center_zstack(IMAGE_STACK, CHANNEL, RESIZE_IMAGE, edge_tol)`

     This function coherently translates a set of z-stacks so that they are (approximately) mean-centered in the frame. It is used for centering entire movies and avoid potential flickering.

   - `IM1 = ROTATE_IMAGE(IM2, R)`

     This function rotates the image im2 using rotation matrix R

   - `images = resize_images(images, npixels)`

     This function resizes a collection of images to the target size defined by npixels.

   - `images = local_normalize_images(images, sigma, epsilon)`

     This function locally normalizes images by average value. At each point, the local average is calculated using the Gaussian kernel of width sigma. The value of the image at that point is then multiplied by $y/(y^2 + \text{epsilon}^2)$.

   - `images = increase_contrast_images(images, a, b)`

     This function increases contrast images through logistic function, $1/(1+\exp(-a*(\text{images}-b)))$.

3. Scattering transform

   - `[Wop, filters] = wavelet_factory_2d(size_in, filt_opt, scat_opt)`

     This function creates a wavelet cascade from morlet filter bank.

   - `S_images = scat_images(images, Wop, ave_angles, pad_factor)`

     This functions computes the scattering transform of the images using the wavelet cascade obtained from wavelet_factory_2d.m

4. Pairwise differences

    - `D = calc_pairwise_distances(images)`
      This function calculates pairwise distances between images using euclidean distance.

5. Diffusion maps (to explore the underlying manifold)

    - `[~, UWighted]=  DiffusionMapsFromDistanceGlobal(d,t,numNeighbors)`
      This function computes diffusion distance and an embedding from a matrix of pairwise distances using the Diffusion Maps algorithm.

6. Affinity matrix

    - `[A,eps] = AffinityFromDistance( d, numNeighbors)`
      This function computes an affinity matrix from given distances. $A_{ij} = \exp(-d_{ij}^2/(\sqrt{\epsilon_i}\sqrt{\epsilon_j}))$, where ϵ_i is a scaling constant around the i-th point and ϵ_i is taken to be the average of the square of the distances from the i-th point to its numNeighbors nearest neighbors. The value of numNeighbors is 10 in our example. This value can be adjusted depending on the density of the sampling of the underlying manifold. The output A is the (n x n) affinity matrix with entries A_{ij}

7. Harmonic extension

    - `[inv_u] = ssl_estimate( W, indl, indu)`
      This function computes the matrix required to obtain the predicted sample values on unlabeled data points from labeled data points. It uses W, the affinity matrix containing the pairwise difference $w_{i,j}$ between all the data points, indl, the indices of the labeled points, indu, the indices of the unlabeled points. The output is inv_u, it is the matrix necessary to compute the predicted labels from the unlabeled ones, f_u = inv_u*Y where Y correspond to the labels.
